# The carbon footprint of steel corrosion

**DOI:** 10.1038/s41529-022-00318-1

**Published:** 2022-12-29

**Authors:** M. Iannuzzi, G. S. Frankel

**Affiliations:** 1 Curtin Corrosion Centre, Curtin University, Perth, Western Australia 6845 Australia; 2 Fontana Corrosion Center, Department of Materials Science and Engineering, The Ohio State University, Columbus, OH 43210 USA

**Keywords:** Policy, Materials science

## Abstract

The monetary cost of corrosion is currently estimated at 3 to 4% of the global GDP considering direct costs exclusively. However, no study to date has quantified the environmental impact associated with steel corrosion. Here, we determined that the CO_2_ emissions associated with the steelmaking required to replace corroded steel will be 4.1–9.1% of the total by 2030 considering the European Union and recent U.S. greenhouse gas reduction targets. We suggest that implementing corrosion management best-practices could drastically reduce the greenhouse gas emissions associated with the replacement of corroded steel and emphasize the need for coordinated international efforts.

## Introduction

Since Uhlig’s 1950 publication^1^, several studies have estimated the economic cost of corrosion. Although the results depend on the methodology and vary from country to country, it is accepted that the direct costs of corrosion to the economy are equivalent to roughly 3–4% of a country’s gross domestic product (GDP)^2,3^. This estimate can likely be applied globally. In this context, the estimates of direct cost group all the measures taken to prevent and manage corrosion. Unlike direct costs, there is no agreement on how to account for the influence of indirect costs, which include the “loss of productivity, compensations for causalities and environmental consequences of corrosion failures, and any other cost that is not directly incurred within that industry”^2^. As a rule of thumb, studies have suggested indirect costs to be equal to direct ones, leading to a total cost of corrosion that could be over 6.2% of the global GDP. According to the World Bank, the global GDP for 2021 was approximately 96.1 trillion US dollars for 2021^4^, resulting in a cost of corrosion of about 6 trillion US dollars. Researchers speculate that between 14 and 33% of the costs could be prevented by implementing current best practices^2^.

While most reports emphasize possible environmental and safety aspects associated with corrosion failures (e.g., leakages, bridge collapses and other hazards), these factors are not considered in their economic models as their costs are difficult to quantify^2^. Moreover, all studies to date have focused on the monetary aspects of the problem, stressing financial losses as a way to persuade stakeholders to take proactive measures to mitigate corrosion.

Even though the negative implications of the monetary cost of corrosion are undeniable, there have been limited attempts to calculate the environmental impact of corrosion as estimated by the carbon dioxide (CO_2_) emissions associated with the manufacturing processes required to replace corroded goods and infrastructure. Recently, Atkins and Lambert quantified the reduction in the so-called carbon dioxide equivalent, CO_2e_, which is an expression of “the total primary energy consumed from direct and indirect processes associated with a product or service”, afforded by different corrosion management strategies under various exposure scenarios^5^. In this regard, the amount of CO_2e_ is proportional to the amount of material lost to corrosion. For example, the amount of CO_2_ associated with steel is roughly equivalent to 1.5 times the mass of the component. Although the reduction in waste CO_2e_ generated by the use of unprotected steel can help identify sustainable corrosion control strategies, the work by Atkins and Lambert does not provide information regarding the aggregated environmental impact of steel corrosion. In this work, we estimate the annual global CO_2_ emissions required to replace steel lost to corrosion and present the result in the context of the 2030 Paris Accord and the recent U.S. climate targets.

## Steel production

**Fig. 1:**
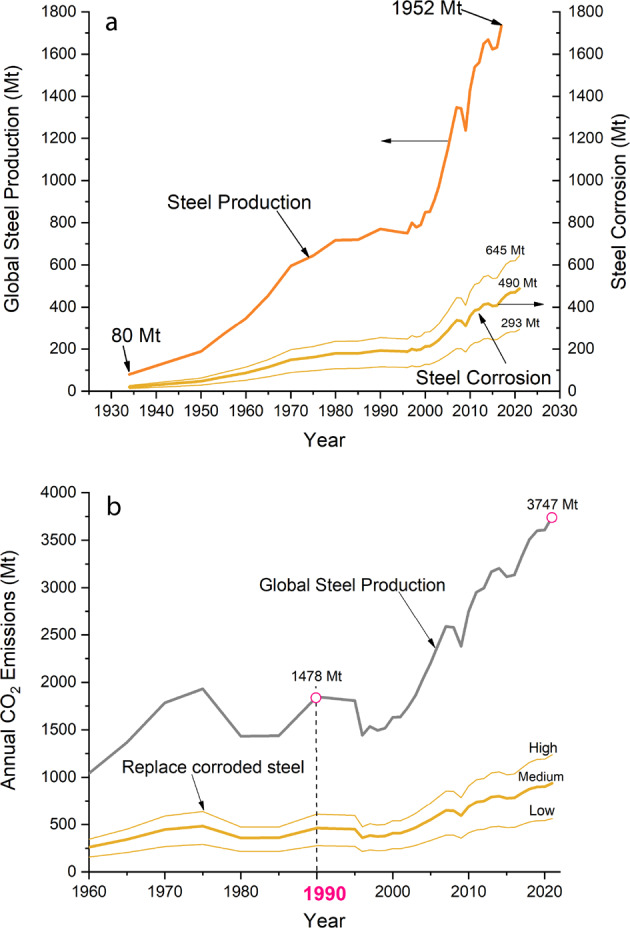
Influence of steel production and corrosion on CO2 emissions. Global steel production and the corresponding amount destined to replace corroded steel (**a**) and the resulting CO_2_ emissions (**b**). The year 1990 is highlighted as it is the bases of the EU target emissions.

In this article, the term *steels* refers to crude iron and steel production, as defined by the World Steel Association^6^. We focus on steels since they are, by volume, the most common engineering metal and are known to corrode in many service environments. Thus, the carbon footprint of corrosion is expected to be dominated by the CO_2_ emissions produced by the steel production process. Figure 1a shows the annual global steel production from 1933 to 2021. The production growth rate steeply increased from the mid-1990s, and it is expected to surpass the 2.5 million tons (Mt) of crude steel per annum by the mid-2020s, despite the global economic slowdown associated with the COVID-19 pandemic. The sharp step-change has been associated with the economic growth in China and India; nevertheless, steel production has expanded globally since the second world war^7^.

Before calculating the carbon footprint of steel corrosion, it is necessary to determine how much of the annual production of crude steel is required to replace corroded goods and infrastructure. Pourbaix and Muylder qualitatively estimated that between 25 and 33% of the annual steel production is destroyed once in service by corrosion^8,9^. These values are somewhat in line with the results of the systematic NBS-Battelle study, which showed that, in the U.S., 17% of the demand of metallic ores in 1978 resulted from metallic corrosion^3^. The figures also correlate well with the energy consumption needed to replace corroded goods, which was 3.4% of the U.S. energy production in 1978^3^. No other cost of corrosion investigation has updated these values.

The estimated percentage of annual steel production that is used to replace steel destroyed by corrosion will directly influence the following analysis. Thus, we propose a range of possible values representing low (15%), medium (25%), and high (33%) scenarios based on the limited data available. Although these ranges are arbitrary, the exact values will not change the primary conclusion of the analysis. Figure 1a also presents the evolution of global steel corrosion, showing that the amount of steel lost to corrosion annually will be in the range of 290 (low)–650 (high) Mt by the mid-2020s.

## CO_2_ emissions to replace corroded steel

Given its high energy intensity, reliance on coal as primary fuel, and the use of CO as a reducing agent, iron and steel production is one of the largest CO_2_ emitters of any industry. Indeed, steel production accounts for 27% of the CO_2_ emissions of the global manufacturing sector, or about 10% of the total global CO_2_ emissions in 2021^10,11^. Due to regulatory pressure on the steel industry driven by environmental concerns, technological advances in steelmaking processes have resulted in the reduction of energy consumption by 61% over the last 50 years^11^. Nevertheless, Kundak et al. argue that only the so-called industrialized countries have made significant advances to improve steel production efficiencies^10^. Likewise, despite promising new refining technologies at different maturity levels^10,12,13^, The World Steel Association estimates that there is little room for further gains with current methods given the vast improvements in energy use^11^.

In this work, we used annualized average carbon dioxide intensity data, defined as carbon dioxide emissions in tons per ton of steel produced (t_CO2_/t_steel_), to estimate CO_2_ emissions per year. Given the marked improvements in energy efficiency, we had to obtain historical carbon dioxide intensity values, which are not readily available prior to the1990s. We used the Indexed Global Energy Consumption per ton of crude steel data to estimate past carbon dioxide intensities^14^. The following parametric carbon intensities were used to simplify the analysis:

- 1960–1975: 3.0 t_CO2_/t_steel_
- 1980–1985: 2.0 t_CO2_/t_steel_
- 1990–1995: 2.4 t_CO2_/t_steel_
- After 1995: 1.9 t_CO2_/t_steel_ (which accounts for the additional 38 Mt_CO2_ produced by the global iron ore mining industry annually)^15^

These figures are supported by the data presented by Birat et al. for France between 1960 and the late 1990s^13^. In their work, the carbon dioxide intensity decreased from 3.6 t_CO2_/t_steel_ in 1960 to a plateau around 1.5 t_CO2_/t_steel_ in 1997. We used an average global carbon dioxide intensity, but there are vast differences in values between countries, which are strongly influenced by the dominant technology used to produce steel^7^.

Figure 1b shows the annual CO_2_ emissions generated by the steel industry and those resulting from steel corrosion. As indicated, the global steel production accounted for almost 3.8 Gt_CO2_ in 2021, of which between approximately 560–1200 Mt_CO2_ could be associated with the replacement of corroded steel. Interestingly, emissions produced in the early 2000s were equivalent to those between 1960 and 1980 despite the much higher annual production (Fig. 1a) due to the marked reduction in carbon dioxide intensity after 1995.

**Fig. 2:**
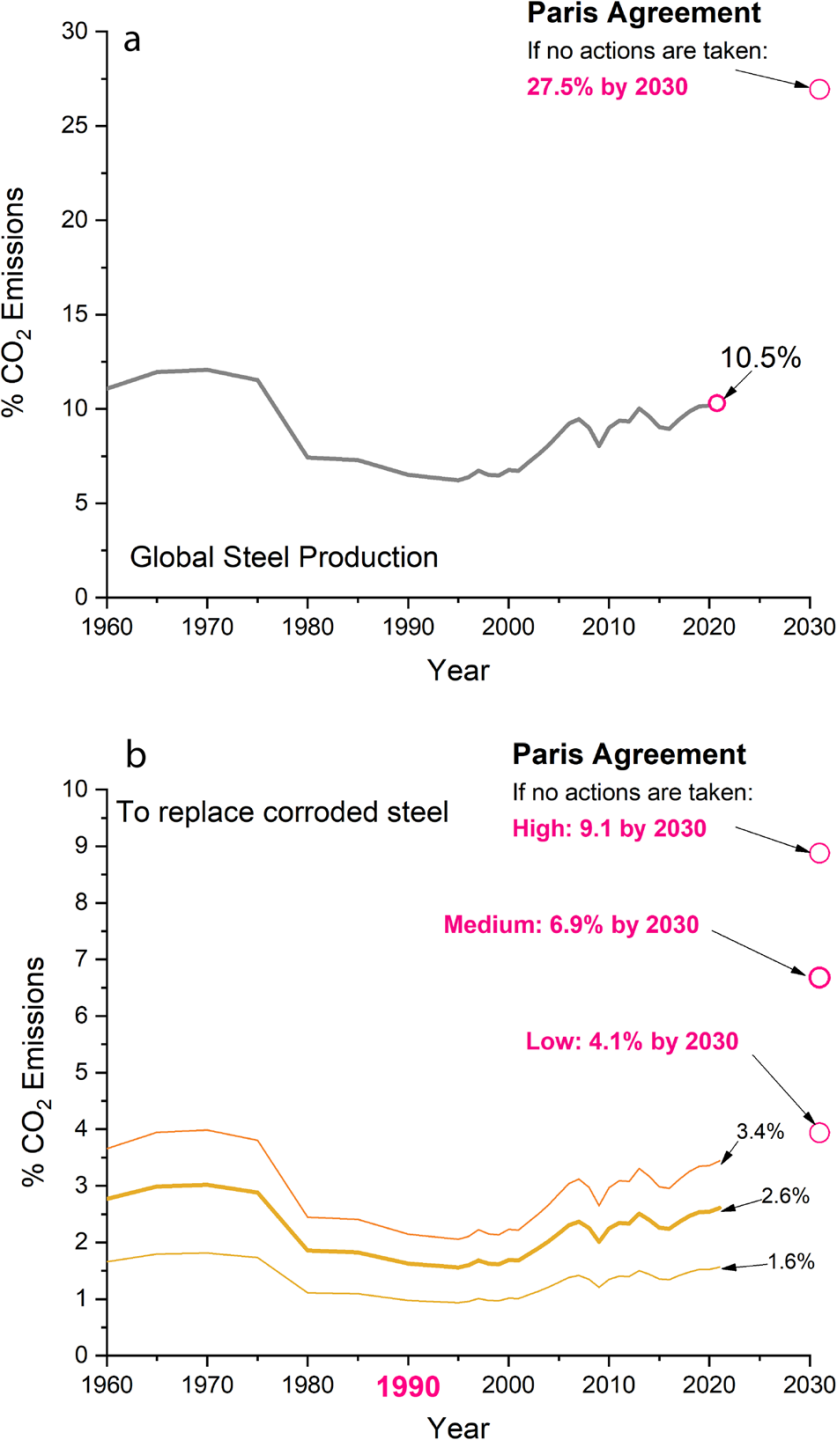
CO2 emissions produced by the steel industry and those destined to replaced corroded steel compared with the 2030 Paris Agreement target. CO_2_ emissions produced by the steel industry as a percentage of the global CO_2_ emissions (%CO_2_) (**a**) and %CO_2_ emissions produced to replace corroded steel (**b**). The year 1990 is highlighted as it is the bases of the EU target emissions.

It is now necessary to contextualize the results shown in (Fig. 1b) as a percentage of the total annual global CO_2_ emissions. Figure 2 shows the total CO_2_ emissions of global steel production and those resulting from the replacement of corroded steel as a percentage of the total global emissions over time. In 2021, steel production represented about 10.5% of the total global CO_2_ emissions, with corroded steel replacement accounting for 1.6–3.4%. Interestingly, the %CO_2_ values in 2021 were lower than those of the 1960s because of the higher carbon dioxide intensities of the older production methods and the much lower overall global CO_2_ emissions.

## The Paris agreement and the new U.S. climate targets

The next step is quantifying how the CO_2_ emissions related to steel production and corrosion compare with accepted target reductions set by nations as part of the Paris Agreement. While these target reductions vary from region to region, it is convenient to evaluate the example set forward by the European Union (EU). In the 2030 Climate & Energy Framework, the EU’s nationally determined contribution under the Paris Agreement is to cut greenhouse gas emissions by at least 55% compared to 1990 levels by 2030^16^. If the EU target reductions were realized globally, the total global CO_2_ emissions would be roughly 13 Gt in 2030^17^. This is in line with the latest U.S. goal promoted by the Biden administration, which mandates a 50-52% reduction in greenhouse gas pollution from 2005 levels by 2030^18^. Considering the U.S. mandate globally, the world’s CO_2_ emissions should be 14.4 to 15 Gt by 2030.

For our calculations, we considered that the total emissions produced by the steel industry remained unchanged from the values from 2021 (i.e., about 2.7 Gt CO_2_ per year), as suggested by the Iron and Steel Technology Roadmap. Despite the predicted steady growth in steel production volumes, the somewhat leveled CO_2_ emissions suggested by the International Energy Agency would be achieved by the adoption of new technologies that reduce carbon dioxide intensities. However, given the sharp rate of increase in global steel demand (Fig. 1a) and the difficulties in the broad global adoption of refining technologies with lower carbon intensities, these assumptions are likely extremely conservative. Therefore, based on the uncertainties in projecting steel production data and the resulting corrosion trends, we considered the approach valid, particularly given the implications discussed below.

As seen in (Fig. 2a), the global CO_2_ emissions of the steel industry would account for 27.5% of the 2030 target values if no actions are taken, with corroded steel replacement representing between 4.1 and 9.1% of the total emissions (Fig. 2b). These results clearly illustrate that the current 2030 targets might be unviable, and that urgent action is needed if the world’s CO_2_ emissions are to be reduced to the values needed to combat climate change. The interpretation of the results depends on the assumptions made, especially those used to define the low, medium, and high values of annual steel production that is used to replace steel destroyed by corrosion. However, reasonably decreasing such values would still lead to similar conclusions. For instance, arbitrarily assuming a very low target corrosion range value of 7% would still result in a considerable 1.9% contribution of corroded steel replacement to the world’s CO_2_ emissions by 2030.

## A call for coordinated international policy

Our goals are to present a new perspective that has been ignored until now and spark debate in the scientific, engineering, and political communities on how to reduce the impact of corrosion on the associated CO_2_ emissions. Addressing this complex problem demands a multifaceted approach combining a large reduction in carbon dioxide intensity, a decrease in the global steel demand, and strict corrosion management policies. Unfortunately, sufficient advancements in reducing carbon dioxide intensities will require radical innovations, e.g., carbon capture and storage, bioenergy, and the use of hydrogen as the reducing agent, which might not gain global traction soon enough^12,19^, and the steel demand does not seem to ease^6^.

However, it might be plausible to reduce carbon footprint of steel production by a proportion equivalent to the monetary savings determined in the various cost of corrosion reports (i.e., 14–33%) by implementing current best practices across industry sectors and countries. Further savings could be realized by adopting new technologies and management strategies that take advantage of advancements in, e.g., big data and machine learning^20^.

In our view, thus, the “no action” scenario is not viable, and we call for a drastic change in corrosion management policies. Failing to act would make meeting the Paris Accord and U.S. target reductions challenging. In this regard, the world’s largest technical association focused on corrosion, NACE International (recently rebranded as AMPP), has proposed a systematic approach that combines expert know-how with economics to combat the cost of corrosion^2^. This approach can address environmental considerations as well as financial aspects.

Analogous to the highly successful Energy Star system and Fuel Economy standards, environmental considerations could be captured in, e.g., a total energy and CO_2_ (TECO_2_) rating. This index should account for the total energy required to produce the material and manufacture the final product, as well as the resulting lifecycle CO_2_ emissions, including those from corrosion. Since TECO_2_ would capture the effect of corrosion on the potential CO_2_ waste associated with a given material-environment combination, TECO_2_ could be used to encourage sustainable materials selection practices. Alternatively, the index could be embedded into current Lifecycle Carbon Accounting practices, communicated to consumers and end users in, e.g., Environmental Product Declarations containing Global Warming Potential metrics. For instance, although the production of costly stainless steels and other corrosion-resistant alloys (CRAs) containing chromium, nickel, and molybdenum, among other alloying elements, is more energy demanding and has higher carbon dioxide intensity values than carbon and low alloy steel production, their service life is usually much longer^21^. As a result, the associated overall CO_2_ emissions of CRAs through the entire structure lifecycle are lower. Thus, in this simplistic example, a corrosion management strategy that partially replaces carbon steel with suitable CRAs would be a preferred approach given the lower TECO_2_ rating and despite the larger capital expenditures. As with the Energy Star and similar international ranking standards, companies could be required to display the TECO_2_ ratings of goods, services, and operations on product labels and social media accounts, which has been proven to drive consumers towards more energy efficient solutions^22^.

Implementing coordinated strategies requires the involvement of policymakers, industry, and academia through coordinated international action. In closing, it should also be emphasized that the estimated CO_2_ emissions associated with corroded steel replacement represent a plausible minimum since we have considered no changes in the projected CO_2_ emissions of the steel industry and ignored the degradation of materials other than steel, e.g., reinforced concrete, non-ferrous alloys, non-metallic materials, and organic coatings. The CO_2_ emissions associated with, e.g., corrosion of reinforced concrete structures could increase the carbon footprint of corrosion substantially, since presently concrete production accounts for about 5% of the global CO_2_ emissions^23^. In our analysis, we have also ignored the emissions of other greenhouse gases that contribute substantially to climate change, e.g., methane, which exacerbates the need for a swift implementation of corrosion control policies to reduce the carbon footprint of corrosion^24^.

## Data Availability

All data are available in the main text and the public domain, as cited.
